# Humidity May Modify the Relationship between Temperature and Cardiovascular Mortality in Zhejiang Province, China

**DOI:** 10.3390/ijerph14111383

**Published:** 2017-11-14

**Authors:** Jie Zeng, Xuehai Zhang, Jun Yang, Junzhe Bao, Hao Xiang, Keith Dear, Qiyong Liu, Shao Lin, Wayne R. Lawrence, Aihua Lin, Cunrui Huang

**Affiliations:** 1 School of Public Health, Sun Yat-sen University, Guangzhou 510080, China; zengj47@mail2.sysu.edu.cn (J.Z.); junzhe_bao@126.com (J.B.); 2 Zhejiang Provincial Center for Disease Control and Prevention, Hangzhou 310051, China; xhzhang@cdc.zj.cn; 3 Institute for Environmental and Climate Research, Jinan University, Guangzhou 510632, China; smart_yjun@163.com; 4 Department of Epidemiology and Biostatistics, School of Public Health, Wuhan University, Wuhan 430072, China; xianghao@whu.edu.cn; 5 School of Public Health, University of Adelaide, Adelaide 5005, Australia; keith.dear@adelaide.edu.au; 6 National Institute for Communicable Disease Control and Prevention, Chinese Center for Disease Control and Prevention, Beijing 102206, China; liuqiyong@icdc.cn; 7 School of Public Health, University at Albany, State University of New York, Albany, NY 12222, USA; slin@albany.edu (S.L.); wlawrence@albany.edu (W.R.L)

**Keywords:** cardiovascular mortality, temperature, relative humidity, joint effect, attributable fraction

## Abstract

*Background*: The evidence of increased mortality attributable to extreme temperatures is widely characterized in climate-health studies. However, few of these studies have examined the role of humidity on temperature-mortality association. We investigated the joint effect between temperature and humidity on cardiovascular disease (CVD) mortality in Zhejiang Province, China. *Methods*: We collected data on daily meteorological and CVD mortality from 11 cities in Zhejiang Province during 2010–2013. We first applied time-series Poisson regression analysis within the framework of distributed lag non-linear models to estimate the city-specific effect of temperature and humidity on CVD mortality, after controlling for temporal trends and potential confounding variables. We then applied a multivariate meta-analytical model to pool the effect estimates in the 11 cities to generate an overall provincial estimate. The joint effects between them were calculated by the attributable fraction (AF). The analyses were further stratified by gender, age group, education level, and location of cities. *Results*: In total, 120,544 CVD deaths were recorded in this study. The mean values of temperature and humidity were 17.6 °C and 72.3%. The joint effect between low temperature and high humidity had the greatest impact on the CVD death burden over a lag of 0–21 days with a significant AF of 31.36% (95% eCI: 14.79–38.41%), while in a condition of low temperature and low humidity with a significant AF of 16.74% (95% eCI: 0.89, 24.44). The AFs were higher at low temperature and high humidity in different subgroups. When considering the levels of humidity, the AFs were significant at low temperature and high humidity for males, youth, those with a low level of education, and coastal area people. *Conclusions*: The combination of low temperature and high humidity had the greatest impact on the CVD death burden in Zhejiang Province. This evidence has important implications for developing CVD interventions.

## 1. Introduction

Environmental risk factors have emerged as a major public health concern, with a growing literature investigating the effects of weather variations on human health. Previous literature has found cardiovascular disease (CVD) is most sensitive to weather across various climates throughout the world [1]. Fueled by economic development, an increasing elderly population, and changes in diet, CVD deaths have increased at alarming rates in developing countries and are predicted to become major causes of morbidity and mortality by 2020 [2]. In China, CVD is predicted to increase by approximately 21.3 million events and 7.7 million deaths over 2010 to 2030 [3,4]. It is imperative to estimate the death burden of CVD associated with environmental risk factors in China.

The Intergovernmental Panel on Climate Change (IPCC) has stated that an increase in extreme weather is the result of climatic change, and one of the greatest challenges we face today worldwide [5]. Numerous studies have demonstrated that extreme temperatures are associated with premature mortality [6]. In general, the temperature–mortality curves were U or V-shaped, with higher risks of mortality in cold and hot weather. Humidity also has significant diurnal and seasonal changes, and is receiving increased attention from a number of health-related analyses [7]. High humidity can reduce the body’s efficiency at transporting away metabolic heat, while low humidity may lead to dehydration [8]. Perspiring is the body’s main physiological response to maintaining core temperature under heat stress. When the air is close to saturation point, this process is inhibited under very humid conditions [9]. Numerous literatures have well explored the independent effects of temperature or humidity on human health [8,10]. However, there are limited studies on their joint effects, particularly in a developing country such as China [9,11,12]. Some previous studies found that the magnitude of effect estimates on mortality for cold temperatures was increased from the north to the south in China [13,14]. The overlooked effect of humidity may result in an underestimation of the consequences of climate change. For this reason, estimating the death burden using the joint effect of temperature and humidity to reduce the death events can be important for human health, especially for cardiovascular health in southern Chinese regions such as Zhejiang Province. Findings can help develop targeted interventions by providing available information and identifying vulnerable populations.

Currently, most studies rely on summaries based on relative risk (RR), odds ratio (OR), and rate ratio, resulting in limited information on excess burden due to volatile weather exposure. The attributable fraction (AF) and attributable number (AN) may solve these problems and could provide the accumulative effect from a specified time period and present the fraction of cases or deaths from a disease [15]. Those indicators are significant in public health and it is recommended that they are used in the health risk assessment of environmental stressors [16].

In this study, we aimed to fill gaps in current knowledge by identifying the effect of relative humidity on the relationship between temperature and CVD mortality. We also identified vulnerable groups and provided more evidence for policymakers to develop public health interventions to reduce the risk effect of CVD mortality.

## 2. Materials and Methods 

### 2.1. Data Collection

**Figure 1:**
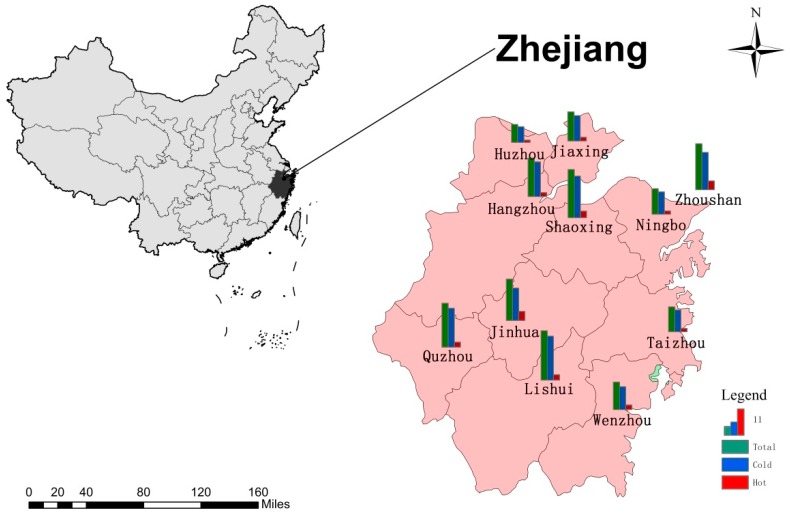
The location of 11 cities in Zhejiang Province and the attributable fraction of cardiovascular disease (CVD) deaths, which were divided into cold and hot temperatures based on distributed lag non-linear model. Cold and hot effects were defined by the minimum mortality temperature. This map was formed by ArcGIS software, Version 10.1, Esri, Redlands, CA, USA.

We collected daily mortality and meteorological data from 11 cities in Zhejiang Province, China. In total, there were seven coastal cities (Hangzhou, Jiaxing, Ningbo, Shaoxing, Taizhou, Wenzhou, and Zhoushan) and four inland cities (Quzhou, Jinhua, Lishui, and Huzhou). Zhejiang Province is located in the southern part of the Yangtze River Delta on the east coast of China with a total land area of 105,500 km^2^ and a population size of 55.9 million in 2016. There are four distinct seasons and overall pleasant climate. The annual mean temperature ranges from 15.0 to 18.0 °C, and the average annual rainfall is 980 to 2000 mm across cities. Figure 1 shows the geographical distribution of the 11 cities in Zhejiang.

The daily count of death data was acquired from the Zhejiang Provincial Center for Disease Control and Prevention during the period 2010 to 2013. The original data included variables on age, sex, education, date of mortality, and cause of death. CVD was based on primary death cause and was categorized by the International Classification of Diseases, Tenth Revision (ICD-10): I00-I99. We conducted stratified analyses by sex, age group (0–74 years and 75+ years), education levels (low: 0–6 years, high: 7+ years), location of cities (coastal and inland), and levels of humidity (low and high of its median value). 

We collected meteorological data from China Meteorological Data Sharing Service System (http://cdc.nmic.cn/home.do). The observation station in each city included daily maximum, minimum, and mean degrees of temperature, relative humidity, wind speed, and atmospheric pressure. The data did not contain any missing values during the study period. Previous studies have documented that atmospheric pressure and wind speed are important risk factors for death. In particular, these two variables may have an influence on humidity [6,17]. We used daily mean temperature and relative humidity to estimate the joint effect between them. The mean temperature indicates the average exposure throughout 24-h average, and the relative humidity is the most commonly used indicator in China and is more easily accepted by the public in their daily life. 

### 2.2. Statistical Analysis

In this study, we applied two-stage analysis strategy. First, we estimated the city-specific effects of temperature on mortality by using time-series regression models. We then stratified analyses of temperature effects by low and high humidity levels. Second, a multivariate meta-regression strategy was used to calculate the overall CVD mortality burden attributable to non-optimal temperatures in different subgroups to identify vulnerable groups [18,19].

For the city-specific analyses, we used time-series Poisson regression within the framework of distributed lag non-linear model to estimate the city-specific association between temperature and CVD mortality after controlling for potential covariates. The model included the following covariates: (1) natural cubic smooth function with 7 degrees of freedom (df) per year to control long-term and seasonal trends; (2) 3 df was used for relative humidity, atmospheric pressure, and wind speed, respectively; (3) days of the week and public holidays were analyzed as categorical variables. Specifically, we modeled the exposure-response curve with a quadratic B-spline with three internal knots placed at the 10th, 75th, and 90th percentiles and the lag-response curve with a natural cubic B-spline with three internal knots placed at equally spaced values in the log scale with maximum lag of 21 days [19,20]. The lag days were extended to 21 days to capture long delay of cold effects and adequately capture the hot effects, as well as harvesting effects. We also estimated temperature effects during the following lag periods: lag 0–3, 0–7 and 0–14 days, respectively. The selection of df and the modeling choices were determined by the Akaike information criterion for quasi-Poisson model (Q-AIC) [21].

For the modifying effect of humidity, we calculated the cold and hot effects on CVD mortality in the stratified analyses by low and high humidity. The minimum mortality temperature (MMT) was used as a reference for calculating the relative risk by re-centering the models. The city-specific cumulative relative risk over lag periods was used to compute the daily attributable death (AD) corresponding to each day’s temperature. The ADs of different levels of humidity were calculated by aggregating the humidity level contributions from the days of the series, and their ratio to the corresponding total number of deaths yielded low or high humidity attributable fraction (AF) [20]. Low and high levels of humidity were based on their median value. The components attributable to cold and hot temperatures were computed by summing the subsets corresponding to days with temperatures below or above the MMT. We calculated the empirical confidence intervals (eCI) using Monte Carlo simulations [19].

In the second stage analysis, we used multivariate meta-analytical model to pool the city-specific effect estimates. We reported both Cochran *Q* test and *I*^2^ statistic to evaluate the heterogeneity of 11 cities. To identify vulnerable subpopulations, we conducted subgroup analyses based on gender, age, education level, and location of cities. 

The sensitivity analyses were performed to check the robustness of the results. We changed the knots of temperature-mortality dimension, the maximum lag days for mean temperature from 14 to 24, and different degrees of freedom per year for time trends from 6 to 10. The df of weather variables such as relative humidity, atmospheric pressure, and wind speed, is from 4 to 6, respectively. All the model choices were performed by Q-AIC. 

All analyses were performed using R software (Version 3.2.2, R Foundation for Statistical Computing, Vienna, Austria) by using DLNM and MVMETA packages to fit the two-stage model. All statistical tests were two-tailed, and *p*-values < 0.05 were considered statistically significant.

## 3. Results

Table 1 shows the descriptive data on daily CVD mortality, by mean temperature, relative humidity, and location. There were 120,544 CVD deaths recorded in 11 cities of Zhejiang Province during 2010 to 2013. There was no statistical significance between-city heterogeneity (*Q* = 48.72, *I*^2^ = 17.9%, *p* = 0.162). The daily mean temperature ranged from −2.7 to 35.7 °C and relative humidity ranged from 17 to 100% in the 11 cities. The median temperature and relative humidity were 18.4 °C and 73%.

Table 2 shows the Spearman correlation between daily CVD death and weather variables. We observed positive correlations between daily CVD death and relative humidity (r = 0.035, *p* < 0.05), and mean temperature and relative humidity (r = 0.018, *p* < 0.05). However, we found negative correlations between daily CVD death and daily mean temperature (r = −0.241, *p* < 0.01). Additionally, the correlations between daily CVD deaths and atmospheric pressure, as well as daily CVD deaths and wind speed, were both significant in this study.

Table 3 shows the meta-analysis of AF for CVD mortality due to the effects of temperature during the following lag periods: 0 to 3, 0 to 7, 0 to 14, and 0 to 21, at different levels of humidity. We observed that hot effects were limited within a few days while the cold effects lasted for 3 weeks (see Supplementary Materials Figures S1 and S2). The AFs were higher at high humidity than low humidity over different lag days. The total AFs were significant on different lag periods and the effects generally increased with more lagged days. When considering the different levels of humidity during different lag period, the highest AF was at low temperature and high humidity with a value of 31.36% (95% eCI: 14.79–38.41%), while the most significant AF was 16.74% (95% eCI: 0.89, 24.44) in the condition of low temperature and low humidity. Supplementary Materials Table S1 shows the estimated AFs individually in each city.

Table 4 shows joint effects of temperature and humidity on CVD mortality by gender, age group, education level, and location of cities. The AFs were higher at low temperature and high humidity in different subgroups. In cold weather and high humidity, the CVD death burden was significant among males, youth, those with a low level of education, and coastal area people, compared with females, elderly, high education, and inland area people. However, we did not observe any significant associations between CVD and hot weather at different levels of humidity.

We conducted sensitivity analyses on modeling choices. The effect estimates for total AFs and QAIC were similar when changing the location of knots for the exposure-lag-response relationship and the degrees of freedom for time and meteorological factors (Supplementary Materials Table S2). The test for the residuals on CVD mortality was approximately independent over time in the model (Supplementary Materials Figure S3). 

## 4. Discussion

To the best of our knowledge, this is the first study to examine whether humidity modifies the association between temperature and CVD mortality in Zhejiang Province, China. The main finding on the joint effects was that low temperature and high humidity could account for majority of CVD death burden, with an AF of 31.36%, and with a significant AF of 16.74% in the condition of low temperature and low humidity. Recently, Gasparrini et al. reported a similar study estimate of 11.3% for all-cause mortality was attributed to ambient temperatures in China, which is smaller than our estimates [19]. A study designed by Yang et al. also reported a similar attributable fraction (17.1%) for CVD mortality due to cold in China [13]. The differences in findings might be due to the different populations and climates. 

Our study showed the impact of temperature on CVD death was higher at high humidity. High humidity may lead to increased thrombotic risk [22], exacerbating the temperature effects on those with existing cardiac health problems. Our study found a positive correlation between daily CVD death and relative humidity (r = 0.035, *p* < 0.05), and a negative correlation between daily CVD death and daily mean temperature (r = −0.241, *p* < 0.01). Similarly, previous studies also found a positive relationship between relative humidity and CVD death, and a positive relationship between temperature and CVD death [23,24]. However, others reported no association between humidity and heart disease, including CVD [23,25]. In general, these contrasting conclusions indicate confounders may potentially influence meteorological variables and disease process. 

Epidemiological studies have verified that susceptibility to extreme weather varies by gender and age [26,27,28]. Our study found that both males and females were at higher risk if exposed to low temperature and high humidity with an AF of 36.55% and 18.70%, and the estimate was significant in males. The present study is supported by a previous study that found the impact of cold temperature on CVD mortality was higher among males and lower during hot weather [29]. This could be due to females usually have a higher percentage of body fat than males, making them more cold-resistant but less heat-resistant; however, this remains unclear with regard to humidity effect [18,30]. 

Our study found youths and the elderly had higher risk at low temperatures and high humidity, with an AF of 32.50% and 27.86%. The CVD death burden was significant and higher among youth when compared to the elderly. Perhaps elderly people are more likely to remain at home and are therefore not exposed to cold temperatures frequently. Moreover, age-appropriate primary care and community-based measures have been targeted for the elderly. In recent years, the proportion of younger men of the total number of employees in industry, construction, and agriculture has been relatively stable in Zhejiang Province, China. Younger people often work outside, resulting in elevated exposure to cold temperatures [30,31,32]. Some studies have also reported that youth are sensitive to the change of humidity on their skin and the sense of touch, and that external irritants disturb their skin’s barrier function [33]. These factors may potentially aggravate the effect of low temperature in youths. Conversely, few studies found that the elderly are especially vulnerable to cold and dry air in other countries [8,18,34]. The most likely explanation is that local geographical conditions and adaptation behaviors are different among these studies. To verify this phenomenon, more data analysis and epidemiological research is warranted in the future.

Poverty is associated with a greater risk of cardiovascular disease, as well as lower levels of education, a major determinant of disease and death in low-income countries [35]. Low education level was found to be significantly associated with cold-humid-related death with an AF of 31.39%. This could be due to poor health, literacy, quality management, control of risk factors, and potentially elevated rates of CVD event and mortality [36]. For these reasons, primary care providers should give more attention to subjects with low levels of education. 

Our study revealed that specific location was associated with cold-related and humid-related deaths. In coastal areas, high temperature and high humidity were associated with higher and more significant risks, with an AF of 27.25% for CVD death. However, we did not observe significant association between joint effect of temperature and humidity on CVD mortality for inland areas. A possible explanation is the air in coastal areas often contains more moisture than inland areas. Majority of residents lived in coastal areas, which resulted in a greater impact on CVD mortality compared with those in inland areas. The frequently adverse weather conditions may cause larger accumulative harmful health impacts to local residents.

Although the mechanism on how humidity affects health remains unclear, it is biologically plausible that the effects of extreme temperature on the cardiovascular system may be involved in the changes in vascular tone, autonomic nervous system response, arrhythmia, and oxidative stress. Cold exposure may raise a number of thrombogenic factors, such as blood cell counts, plasma cholesterol, C-reactive protein, fibrinogen concentrations, and platelet reactivity. It could also increase the frequency of heart rate and ventricular ectopic beats, and may contribute to morbidity and mortality in sympathetic nervous system and the hemodynamic system when responding to cold stress [37]. Meanwhile, exposure to hot weather may increase blood viscosity and cardiac output, which lead to dehydration, hypotension, surface blood circulation increase, and even endothelial cell damage [38,39]. Humidity is linked with anomalous mortality and morbidity, because it affects cold or heat stress and hydration state [40]. During extreme temperature and humidity, the body is under considerable constraint from a range of stress-related physiological reactions [41]. The potential impacts on cardiac function from high body-core temperatures and low hydration levels can be related to atmospheric moisture state, increased cardiac function, or work. The underlying mechanisms are unclear and require additional investigation [42].

The present study makes a number of contributions, as follows: To the best of our knowledge, this is the first study to provide comprehensive evidence that the joint effect of temperature and humidity is an important factor in CVD mortality in China. Specifically, majority of previous studies were based on relative risk and other diseases, and did not consider the integrated effects of temperature and humidity on CVD mortality [6,43]. Furthermore, our study is based on a multicenter analysis combining the pooled effect estimates, which include large numbers of CVD deaths, providing sufficient statistical power to examine the joint effect between temperature and humidity. A multicenter study would benefit from a large multicity data set, in order to make the result more stable.

Our present study also has limitations. First, data were only from 11 cities in Zhejiang province. For this reason, it is important to be cautious when extending our findings to other areas in China because of the unique weather conditions and other city-specific parameters. Second, measurement errors with temperature and humidity are inevitable and the collected data from monitoring stations may not best reflect real exposure level, especially for those persons who died far from the station. Third, we have only considered the deaths where CVD was the underlying cause. To provide greater knowledge, we would need to separately investigate people with and without preexisting CVD. Fourth, the data on air pollution were unavailable, and unmeasured residual factors such as smoking, family history of CVD, air condition use, and activity patterns may potentially confound the relationship.

The significantly influential study should play an important role in public health policy implications. First, our findings suggest identified susceptible subpopulations that should be specifically targeted. Second, our analysis found that humidity is a modifier in southern areas on human health that can be extended to other areas in further studies. Third, this study could encourage health care institutions and governmental agencies to establish a fair policy on climate change adaptation. 

## 5. Conclusions

Our study found that humidity might be an important factor to modify the relationship between temperature and mortality in Zhejiang, China. This was also observable for the joint effect between low temperature and high humidity, having the greatest influence on CVD death burden, in which the effect was significant and stable at different lag periods. At different levels of humidity, the AFs were highest at low temperature and high humidity in stratified analyses by gender, age group, education level, and location of cities. In addition, the effects were significant for males, youth, those with a low level of education, and individuals in coastal areas. These identified, susceptible subpopulations should be specifically protected. Evidence from our study is complementary to the literature and has important public health implications for planning intervention measures to reduce CVD mortality risk. Further research needs to be verified and extended to other areas.

## Figures and Tables

**Table 1:** Summary statistics for CVD deaths, daily mean temperature, and relative humidity in Zhejiang Province, China between 2010 and 2013.

City	Total CVD Deaths	Variable	Mean	Min	P25	P50	P75	Max
Hangzhou	14,376	Humidity	69.7	17.0	59.0	71.0	82.0	97.0
		Temperature	17.5	−2.0	9.8	18.3	25.6	35.7
Huzhou	8765	Humidity	73.9	26.0	67.0	76.0	82.0	99.0
		Temperature	16.9	−1.5	9.6	18.0	24.3	32.3
Jiaxing	11,793	Humidity	73.7	31.0	66.0	74.0	82.0	98.0
		Temperature	17.1	−2.7	9.0	18.1	24.7	35.0
Jinhua	13,730	Humidity	66.5	22.0	57.0	66.0	77.0	96.0
		Temperature	18.2	−1.4	10.8	19.2	25.9	35.6
Lishui	6098	Humidity	69.6	26.0	61.0	70.0	79.0	96.0
		Temperature	18.6	−0.3	11.7	19.4	26.0	34.2
Ningbo	13,037	Humidity	72.0	19.0	64.0	73.0	81.0	95.0
		Temperature	17.5	−1.6	9.7	18.5	25.2	34.4
Quzhou	5026	Humidity	72.9	36.0	64.0	72.0	83.0	98.0
		Temperature	17.7	−1.5	10.4	18.5	25.4	34.7
Shaoxing	14,815	Humidity	72.5	21.0	63.0	73.0	83.0	97.0
		Temperature	17.1	−2.3	9.6	17.9	25.1	34.9
Taizhou	14,782	Humidity	73.9	20.0	67.0	75.0	82.0	100.0
		Temperature	17.7	0	10.5	18.4	25.3	33.0
Wenzhou	15,875	Humidity	74.6	26.0	67.0	76.0	84.0	99.0
		Temperature	18.6	0.7	11.7	19.2	25.9	32.2
Zhoushan	2247	Humidity	75.5	28.0	66.0	78.0	86.0	97.0
		Temperature	16.3	−1.8	9.2	17.4	23.2	31.8
Overall	120,544	Humidity	72.3	17.0	64.0	73.0	82.0	100.0
		Temperature	17.6	−2.7	10.2	18.4	25.1	35.7

Note: P25, P50, P75 are the 25th, 50th, 75th percentile of daily mean temperature and relative humidity, respectively; Min: minimum; Max: maximum.

**Table 2:** Spearman’s correlation between daily CVD deaths and weather variables.

	CVD Deaths	Mean Temperature	Relative Humidity	Atmospheric Pressure	Wind Speed
CVD deaths	1.000				
Mean temperature	−0.241 **	1.000			
Relative humidity	0.035 *	0.018 *	1.000		
Air pressure	0.323 **	−0.801 **	−0.176 **	1.000	
Wind speed	−0.223 **	−0.011	−0.047 **	−0.061 **	1.000

Note: ** *p* < 0.01, * *p* < 0.05.

**Table 3:** The pooled attributable fractions of cold and hot effects on CVD mortality over multiple lag days at different levels of humidity.

Lag Days	Humidity Level	Attributable Fraction (%, 95% Empirical CI)		
		Total	Cold	Hot
0–3	Low-humidity	9.30 (3.77, 12.94)	4.32 (−0.14, 7.70)	4.99 (2.27, 7.15)
	High-humidity	11.80 (5.40, 16.32)	6.55 (2.57, 9.47)	5.24 (0.46, 8.56)
0–7	Low-humidity	11.63 (5.08, 15.68)	7.59 (1.45, 11.76)	4.04 (1.07, 6.24)
	High-humidity	21.40 (12.03, 26.85)	16.01 (8.52, 20.52)	5.39, 0.51, 8.50)
0–14	Low-humidity	13.11 (6.58, 17.36)	9.96 (3.69, 14.11)	3.15 (0.56, 5.19)
	High-humidity	25.59 (11.41, 33.12)	20.34 (9.54, 25.69)	5.26 (−3.29, 10.36)
0–21	Low-humidity	19.18 (3.43, 25.64)	16.74 (0.89, 24.44)	2.44 (−0.62, 4.38)
	High-humidity	34.28 (12.93,41.56)	31.36 (14.79,38.41)	2.92 (−6.54, 7.70)

**Table 4:** The pooled attributable fractions of cold and hot effects on CVD mortality over lag 0–21 days at different levels of humidity.

	Humidity Level	Attributable Fraction (%, 95% Empirical CI)		
		Total	Cold	Hot
Male	Low-humidity	25.98 (10.98, 32.68)	24.65 (9.01, 31.13)	1.34 (−1.04, 2.59)
	High-humidity	39.24 (24.66, 48.23)	36.55 (21.62, 45.33)	2.69 (−2.10, 4.73)
Female	Low-humidity	21.83 (−12.61, 28.76)	14.81 (−6.57, 21.93)	7.02(−10.12, 12.63)
	High-humidity	34.58 (−12.32, 40.23)	18.70 (−16.99, 30.72)	15.88 (−12.98, 22.63)
Age 0–74	Low-humidity	32.86 (−16.88, 31.42)	23.23 (5.95, 28.79)	9.63 (−13.94, 25.42)
	High-humidity	37.83 (−12.75, 45.22)	32.50 (2.87, 40.12)	5.23 (−10.22, 15.32)
Age 75+	Low-humidity	24.89 (−9.15, 32.85)	20.74 (−10.13, 28.88)	4.15 (−1.23, 7.05)
	High-humidity	33.42 (−4.10, 42.56)	27.86 (−5.86, 37.48)	5.56 (−8.71, 10.55)
Low-Education	Low-humidity	18.15 (−10.91, 27.95)	15.84 (−16.58, 25.71)	2.30 (−0.37, 4.02)
	High-humidity	33.06 (3.81, 41.37)	31.39 (5.54, 40.14)	1.67 (−2.64, 3.83)
High-Education	Low-humidity	32.01 (−18.23, 43.22)	29.06 (−7.32, 45.33)	2.94 (−5.27, 4.51)
	High-humidity	38.69 (−14.73, 47.33)	33.82 (−10.22, 36.55)	4.87 (−5.82, 13.35)
Coastal	Low-humidity	23.13 (2.91, 31.32)	19.91 (1.43, 29.97)	3.22 (−2.42, 6.31)
	High-humidity	33.85 (19.04, 42.29)	27.25 (18.33, 35.14)	6.59 (−6.78, 12.06)
Inland	Low-humidity	22.13 (−8.53 ,35.54)	15.58 (−19.33, 27.86)	6.55 (−0.76, 12.32)
	High-humidity	30.79 (−12.77, 40.67)	29.25 (−12.87, 39.22)	1.55 (−2.87, 3.16)

## Supplementary Materials

The following are available online at www.mdpi.com/1660-4601/14/11/1383/s1. Table S1: The attributable fractions of CVD mortality due to cold and hot effects over lag 0–21 days stratified by low and high levels of humidity in 11 cities of Zhejiang province, China; Table S2: Sensitivity analyses of attributable fractions for CVD mortality due to cold and hot effects by changing knots, lag and degrees of freedom (df) for the model; Figure S1: The lag-response relation associated with cold temperature (2.5th percentile versus minimum-mortality temperature) over lag 0–21 days on CVD mortality in 11 cities of Zhejiang province; Figure S2: The lag-response relation associated with hot temperatures (97.5th percentile versus minimum-mortality temperature) over lag 0–21 days on CVD mortality in 11 cities of Zhejiang province; Figure S3: The residual variation scatter plots over time for main model in daily deaths after controlling seasonal and long-term trend of 11 cities in Zhejiang province.
