# Supplementary material for: Humidity May Modify the Relationship between Temperature and Cardiovascular Mortality in Zhejiang Province, China
Source: Int J Environ Res Public Health. 2017 Nov 14;14(11):1383. doi: 10.3390/ijerph14111383 (PMC5708022; doi:10.3390/ijerph14111383)
Supplement: Supplementary file 1 [file ijerph-14-01383-s001.pdf]

International Journal of
Environmental Research and Public Health

MDPI

Supplemental Materials: Humidity may modify the relationship between temperature and cardiovascular mortality in Zhejiang Province, China

Jie Zeng [1,†], Xuehai Zhang [2,†], Jun Yang [3], Junzhe Bao [1], Hao Xiang [4], Keith Dear [5], Qiyong Liu [6], Shao Lin [7], Wayne R. Lawrence [7], Aihua Lin [1,*] and Cunrui Huang [1,*]

OUTLINE

Title	Page
Supplementary Table S1: The attributable fractions of CVD mortality due to cold and hot effects over lag 0-21 days stratified by low and high levels of humidity in 11 cities of Zhejiang province, China.	2
Supplementary Table S2: Sensitivity analyses of attributable fractions for CVD mortality due to cold and hot effects by changing knots, lag and degrees of freedom (df) for the model.	3
Supplementary Figure S1: The lag-response relation associated with cold temperature (2.5th percentile versus minimum-mortality temperature) over lag 0-21 days on CVD mortality in 11 cities of Zhejiang province.	4
Supplementary Figure S2: The lag-response relation associated with hot temperature (97.5th percentile versus minimum-mortality temperature) over lag 0-21 days on CVD mortality in 11 cities of Zhejiang province.	5
Supplementary Figure S3: The residual variation scatter plots over time for main model in daily deaths after controlling seasonal and long-term trend of 11 cities in Zhejiang province.	6

Table S1. The attributable fractions of CVD mortality due to cold and hot effects over lag 0-21 days stratified by low and high levels of humidity in 11 cities of Zhejiang province, China.

City	Humidity level	MMT(°C)	Attributable fraction(%,95%empirical CI)		
			Total	Cold	Hot
Hangzhou	Low-humidity	32.70	18.06 (-35.64,44.28)	17.82 (-36.87,45.33)	0.25 (-1.18,1.03)
	High-humidity	30.19	24.77 (-42.84,30.77)	24.88 (-40.05,31.11)	-0.11 (-0.87,0.22)
Huzhou	Low-humidity	29.20	31.03 (-27.14,49.43)	30.47 (-30.20,49.89)	0.56 (-0.30,1.02)
	High-humidity	29.92	40.21 (-17.56,50.21)	40.11 (-15.68,52.11)	-0.11(-2.03,0.41)
Jiaxing	Low-humidity	33.54	38.18 (-17.13,40.58)	38.15 (-20.65,44.59)	0.03 (-0.64,0.44)
	High-humidity	30.00	40.14 (-18.28,51.22)	39.92 (-3.21,48.22)	-0.22 (-0.88,0.09)
Jinhua	Low-humidity	18.30	14.70 (-4.21,45.03)	7.47 (-4.48,36.80)	7.22 (-11.49,18.78)
	High-humidity	14.20	10.79 (-20.11,30.11)	1.88 (-1.02,3.69)	8.92 (-21.22,13.21)
Lishui	Low-humidity	28.28	23.69 (-29.97,48.92)	19.58 (-35.82,45.47)	4.10 (-1.33,7.43)
	High-humidity	14.84	27.67 (-12.12,40.22)	11.70 (-1.55,20.32)	15.97 (-20.72,30.47)
Ningbo	Low-humidity	18.12	13.65 (2.28,20.87)	9.51 (-3.07,17.99)	4.13 (-7.60,12.26)
	High-humidity	29.90	30.22 (-8.95,40.22)	30.41 (3.57,39.99)	-0.19 (-0.59,0.06)
Quzhou	Low-humidity	17.67	13.95 (-1.22,22.54)	7.10 (-2.29,13.67)	6.84 (-12.84,19.04)
	High-humidity	28.90	28.42 (-12.22,40.22)	28.56 (-14.33,38.23)	-0.14 (-0.83,0.20)
Shaoxing	Low-humidity	31.42	17.42 (-24.65,41.64)	16.64 (-25.13,39.45)	0.78 (-0.82,1.70)
	High-humidity	29.61	35.22 (8.01,40.22)	35.48 (6.80,42.12)	-0.26 (-1.13,0.13)
Taizhou	Low-humidity	30.12	11.77 (-38.96,41.79)	11.11 (-43.36,42.05)	0.66 (-1.06,1.78)
	High-humidity	29.23	25.33 (-17.21,29.12)	25.14 (-17.16,30.23)	0.18 (-2.25,0.97)
Wenzhou	Low-humidity	23.40	15.79 (-36.41,40.41)	12.59 (-34.37,38.92)	3.20 (-7.04,9.23)
	High-humidity	14.27	6.14 (-12.21,22.40)	1.50 (-13.73,10.35)	4.64 (-10.27,15.22)
Zhoushan	Low-humidity	27.19	23.48 (-10.32,30.80)	20.82 (-10.22,30.21)	2.66 (-0.23,4.00)
	High-humidity	28.60	32.22 (12.96, 42.11)	32.36 (4.28,40.18)	-0.14 (-5.65,0.38)
Overall	Low-humidity	28.28	19.18 (3.43,25.64)	16.74 (0.89,24.44)	2.44 (-0.62,4.38)
	High-humidity	29.23	34.28 (12.93,41.56)	31.36 (14.79,38.41)	2.92 (-6.54,7.70)

Note: MMT, minimum-mortality temperature

Table S2. Sensitivity analyses of attributable fractions for CVD mortality due to cold and hot effects by changing knots, lag and degrees of freedom (df) for the model.

Mo Model Choice	Attributable fraction(%,95%empirical CI)			QAIC
	Total	Cold	Hot	
**Reference**	28.05 (21.94,32.38)	27.47(21.77,31.81)	0.58 (0.19,0.91)	**4552.10**
Knots for exposure-response: 25th- 50th and 75th	23.03 (16.34,27.30)	22.19 (15.75,26.51)	0.83 (-0.70,1.99)	4559.45
Knots for exposure-response: 25th- 75th and 90th	26.94 (20.31,31.00)	26.15 (19.50,30.39)	0.79 (-0.18,1.36)	4557.76
Lag period: 14 days	22.51 (16.16,26.92)	21.80 (15.55,26.34)	0.71 (0.37,0.98)	4575.61
Lag period: 20 days	27.90 (21.56,32.04)	27.27 (20.79,31.62)	0.63 (0.19,0.95)	4563.59
Lag period: 24 days	25.79 (18.20,30.70)	25.11 (17.59,30.18)	0.68 (-0.13,1.19)	4552.14
Df for year: 6	21.77 (16.06,25.93)	21.35 (15.16,25.61)	0.42 (-0.07,0.74)	4556.85
Df for year: 8	23.46 (16.07,28.49)	22.45 (14.89,27.67)	1.00 (-0.14,1.76)	4570.85
Df for year: 10	20.65 (8.85,26.90)	18.42 (8.05,23.79)	2.23 (-2.60,4.70)	4574.70
Df for relative humidity: 4	28.10 (21.72,32.34)	27.52 (21.29,31.80)	0.59 (0.17,0.92)	4564.21
Df for relative humidity: 6	28.10 (22.08,32.15)	27.51 (21.08,31.65)	0.59 (0.16,0.91)	4568.03
Df for air pressure: 4	28.10 (22.33,32.43)	27.51 (21.64,31.72)	0.59 (0.17,0.94)	4553.34
Df for air pressure : 6	27.67 (21.20,31.67)	26.88 (20.84,31.11)	0.58 (0.19,0.89)	4557.63
Df for wind speed: 4	27.81 (21.64,31.73)	27.22 (20.63,31.57)	0.59 (0.18,0.93)	4557.93
Df for wind speed: 6	27.62 (21.52,31.86)	27.04 (20.71,31.02)	0.59 (0.19,0.87)	4555.96

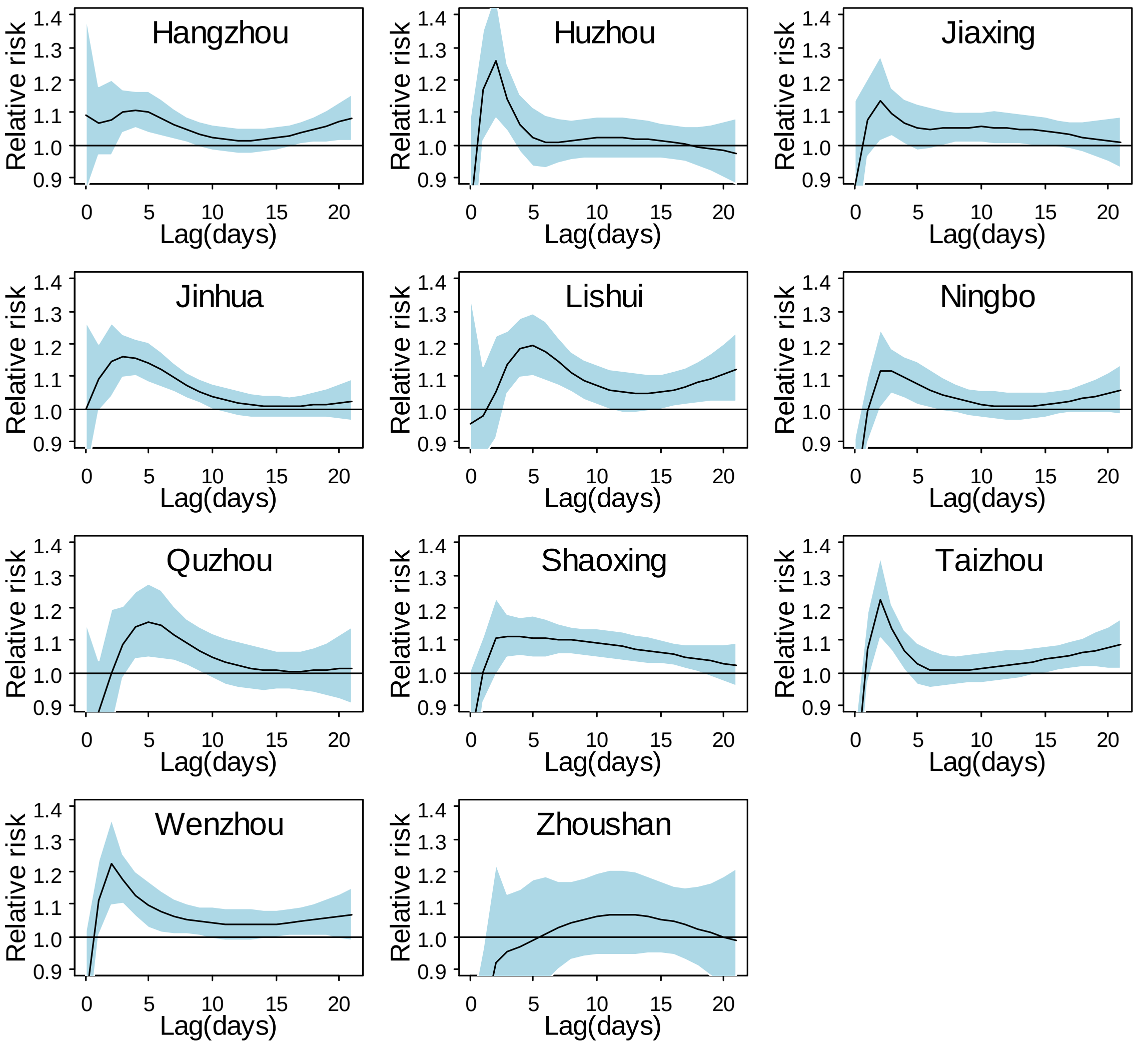

Figure S1. The lag-response relation associated with cold temperature (2.5th percentile versus minimum-mortality temperature) over lag 0–21 days on CVD mortality in 11 cities of Zhejiang province.

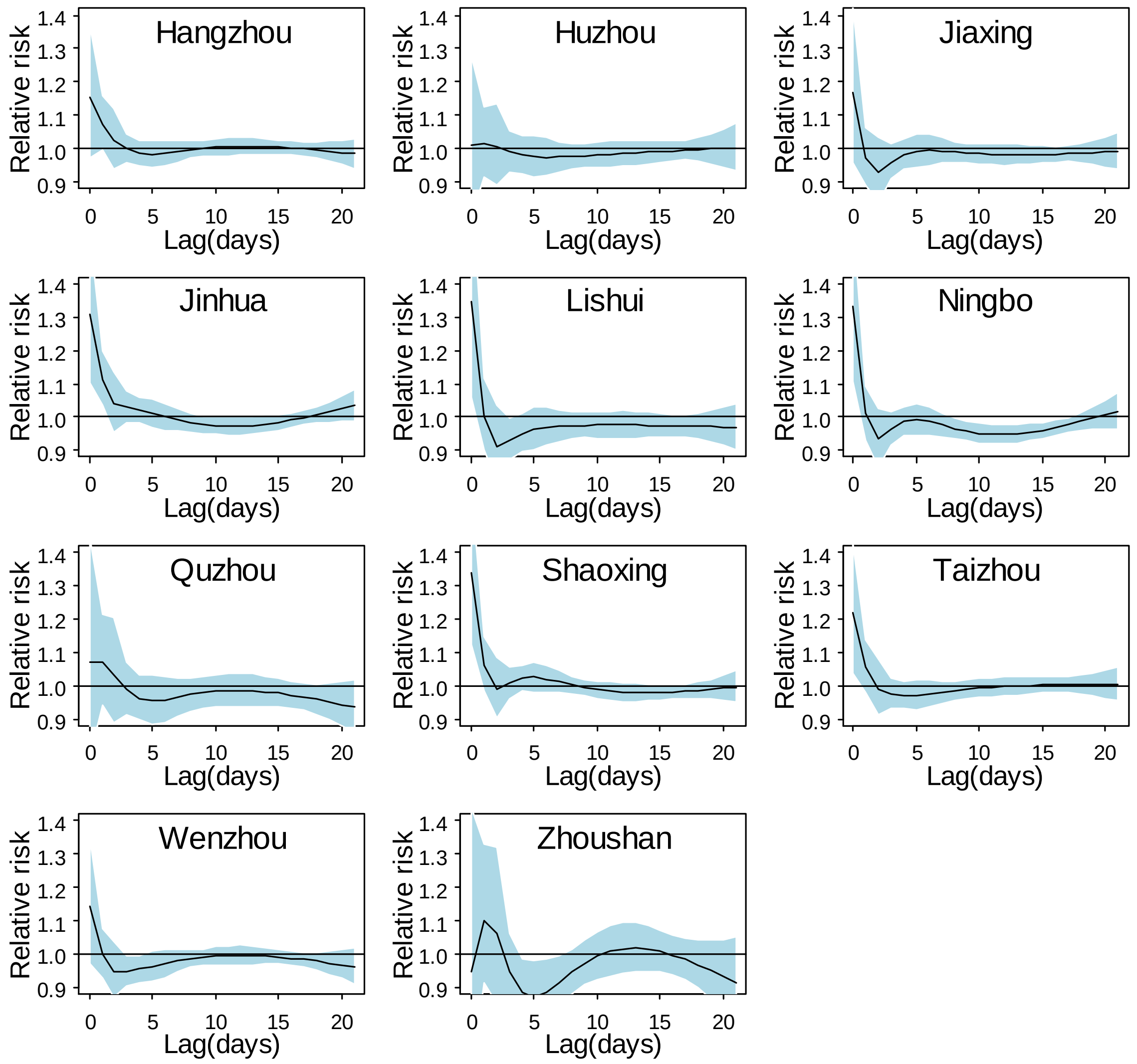

Figure S2. The lag-response relation associated with hot temperature (97.5th percentile versus minimum-mortality temperature) over lag 0–21 days on CVD mortality in 11 cities of Zhejiang province.

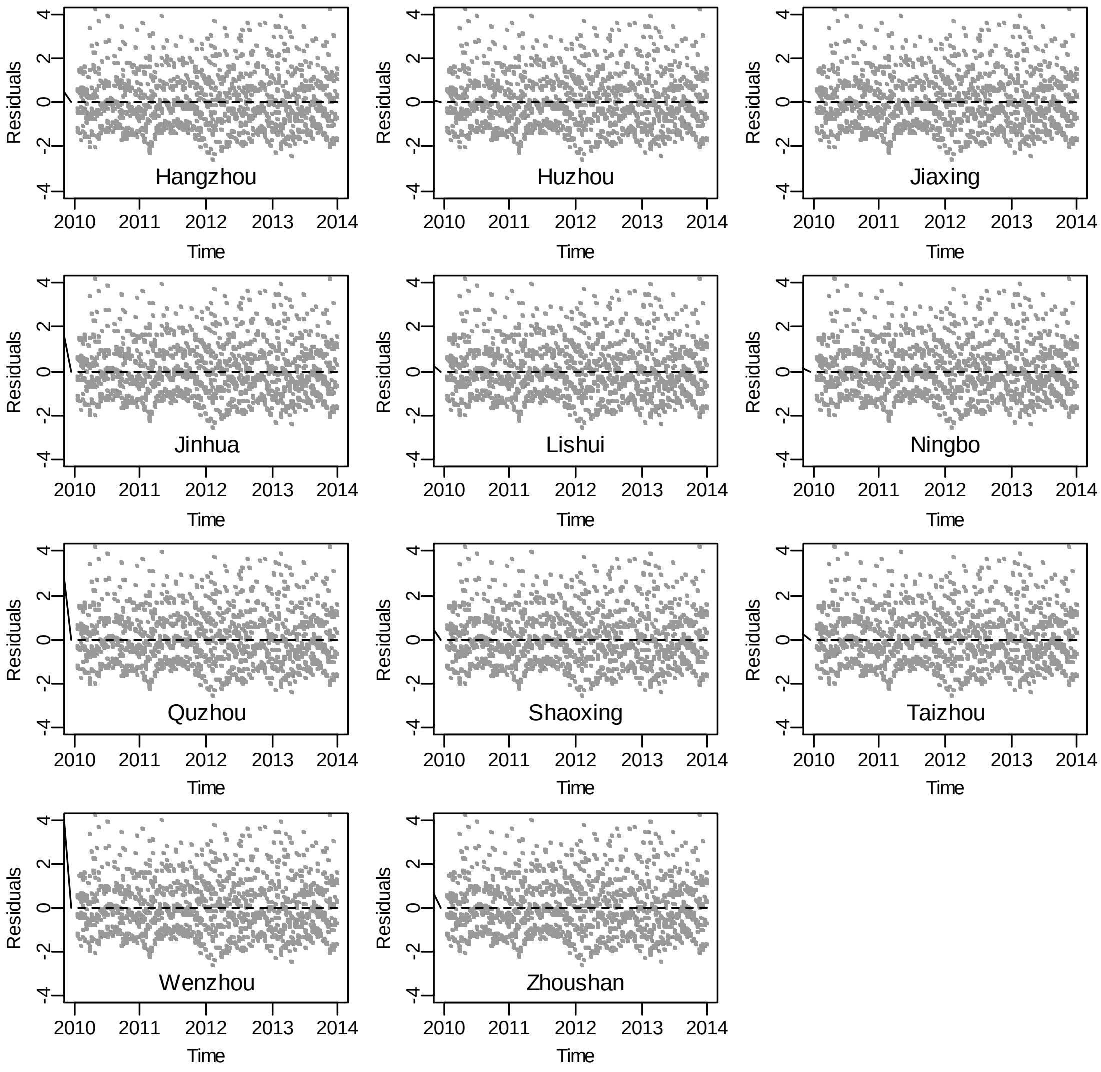

Figure S3. The residual variation scatter plots over time for main model in daily deaths after controlling seasonal and long-term trend of 11 cities in Zhejiang province.

© 2017 by the authors. Submitted for possible open access publication under the terms and conditions of the Creative Commons Attribution (CC BY) license (http://creativecommons.org/licenses/by/4.0/).
